# Machine learning to predict mortality after rehabilitation among patients with severe stroke

**DOI:** 10.1038/s41598-020-77243-3

**Published:** 2020-11-18

**Authors:** Domenico Scrutinio, Carlo Ricciardi, Leandro Donisi, Ernesto Losavio, Petronilla Battista, Pietro Guida, Mario Cesarelli, Gaetano Pagano, Giovanni D’Addio

**Affiliations:** 1 Istituti Clinici Scientifici Maugeri IRCCS, Pavia, Italy; 2 Department of Advanced Biomedical Sciences, University Hospital of Naples “Federico II”, Naples, Italy; 3 Department of Electrical Engineering and Information Technology, University of Naples “Federico II”, Naples, Italy

**Keywords:** Neurology, Engineering

## Abstract

Stroke is among the leading causes of death and disability worldwide. Approximately 20–25% of stroke survivors present severe disability, which is associated with increased mortality risk. Prognostication is inherent in the process of clinical decision-making. Machine learning (ML) methods have gained increasing popularity in the setting of biomedical research. The aim of this study was twofold: assessing the performance of ML tree-based algorithms for predicting three-year mortality model in 1207 stroke patients with severe disability who completed rehabilitation and comparing the performance of ML algorithms to that of a standard logistic regression. The logistic regression model achieved an area under the Receiver Operating Characteristics curve (AUC) of 0.745 and was well calibrated. At the optimal risk threshold, the model had an accuracy of 75.7%, a positive predictive value (PPV) of 33.9%, and a negative predictive value (NPV) of 91.0%. The ML algorithm outperformed the logistic regression model through the implementation of synthetic minority oversampling technique and the Random Forests, achieving an AUC of 0.928 and an accuracy of 86.3%. The PPV was 84.6% and the NPV 87.5%. This study introduced a step forward in the creation of standardisable tools for predicting health outcomes in individuals affected by stroke.

## Introduction

Stroke is among the leading causes of death and disability worldwide^1–4^. Approximately 20–25% of stroke survivors present severe disability^5^. Severe disability after stroke is associated with increased risk of mortality and readmission, wider inter-individual variation in responsiveness to rehabilitation, and higher healthcare and social costs compared with less severe strokes^6,7^. Moreover, there is evidence that patients with severe post-stroke disability are less likely to be admitted to specialized inpatient rehabilitation facilities (IRF) and to receive appropriate secondary prevention than those with mild-to-moderate disability^8–12^, with a possible negative impact on prognosis.

Prognostication is inherent in the process of clinical decision-making^13^. The assessment of risk in stroke patients with severe disability might improve clinical decision-making, prompt clinicians to consider closer surveillance and more aggressive treatment to achieve goals in secondary prevention, and influence patient management. While not routinely used in clinical practice, multivariable models are well-accepted tools to predict prognosis. Three well-known prognostic models were developed to predict 90-day or 1-year mortality in patients with acute stroke^14–16^. These models had good discriminatory properties (C statistic ranging 0.706 and 0.840). However, the application of models developed from patients with heterogeneous neurological deficits using variables recorded at acute care admission to the subset of patients with severe stroke after discharge from the acute care setting can result in miscalibrated estimates of life expectancy and decreased discriminatory value. In addition, the beneficial effect of inpatient rehabilitation on mortality might confound the association between predictors recorded at admission to acute care and mortality^17–19^.

The standard approach to develop prognostic models involves the use of statistical regression models. Correlation between covariates, nonlinearity of the association between continuous covariates and risk for the outcome of interest, and potential complex interactions among covariates represent common analytic challenges in regression modelling^20,21^. In comparison with statistical models, machine-learning (ML) methods have the advantages of using a larger number of predictors, requiring fewer assumptions, using an agnostic approach instead of a priori hypotheses, incorporating “multi-dimensional correlations that contain prognostic information”, and producing a “more flexible relationship among the predictor variables (alone or in combination) and the outcome”^20,22–24^. As observed by Deo^24^, “there may be features that are useful in combinations but not on their own”. Theoretically, these properties might allow achieve an improved model performance for prognostication of the outcome of interest.

**Figure 1:**
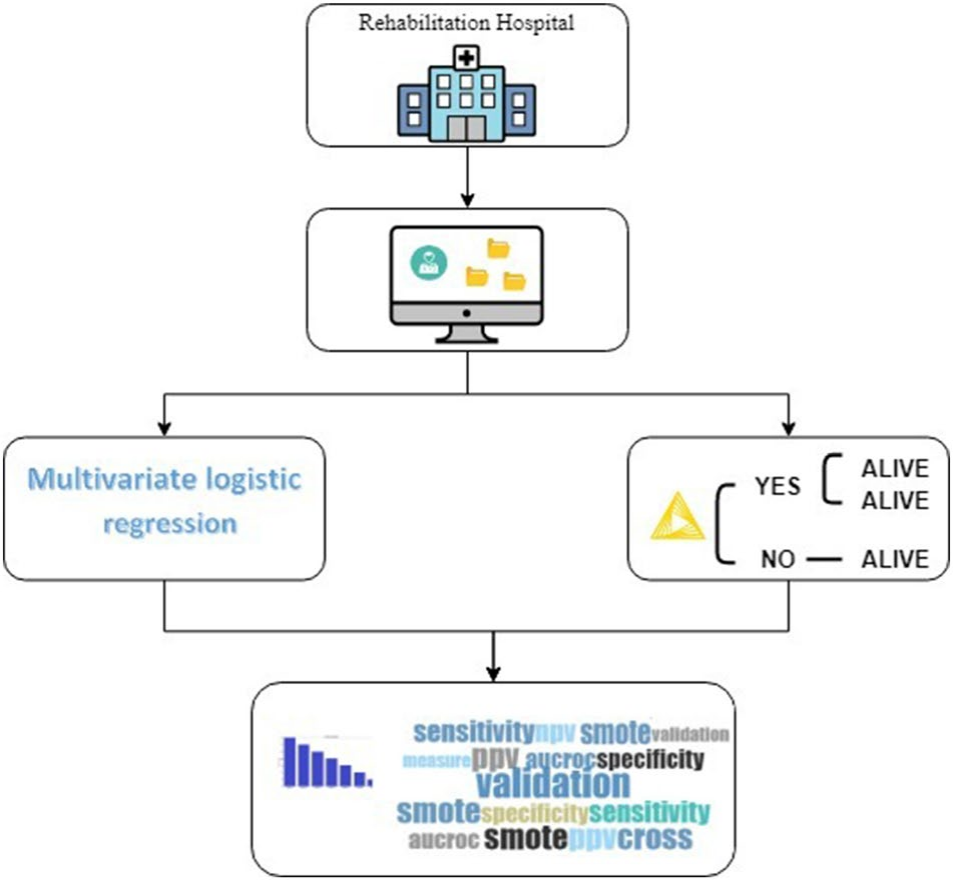
The workflow of the study is represented: the data of 1207 patients from three facilities of Maugeri Institute in the South and in the North of Italy were collected and used to create models through a multivariate logistic regression and tree-based ML algorithms to predict three-year mortality in stroke patients after rehabilitation.

The workflow of the study is shown in Fig. 1 and its aim was two-fold:

1. Assessing the performance of ML–based algorithms for predicting long-term mortality in stroke patients with severe disability;
2. Comparing the performance of ML algorithms to that of a standard regression model.

To address these issues, we studied 1207 patients admitted to inpatients rehabilitation and classified as Case-Mix Groups (CMGs) 0108, 0109, and 0110 of the Medicare case-mix classification system^25^, which was specifically developed to account for “the level of severity of a given case”^26^. Case-mix groups 0108, 0109, and 0110 encompass the most severe strokes. Since our primary was a dichotomous outcome (dead/alive) rather than time-to-event and nearly all survivors had a complete follow-up up to three years, we chose to focus on a logistic regression analysis instead of a Cox regression analysis. We found that ML algorithms outperformed a standard regression model.

## Results

Table 1 shows baseline patients’ characteristics. Of the 1241 patients who fulfilled the selection criteria, 34 were lost to follow-up after discharge, leaving 1207 patients available for analysis. A total of 3,267 person-years of follow-up were examined during which 189 deaths (5.8 deaths/100 person-years) occurred. The mean follow-up was 988 ± 273 days. The actual mortality rates were 8.3% at 1 year, 13.0% at 2 years, and 15.7% at 3 years.

**Table 1:** Baseline characteristics.

**Demographics**		
Age (years), mean (SD)	71 (12)	
< 65 years, n (%)	289 (23.9)	
65 to 74 years, n (%)	348 (28.8)	
≥ 75 years, n (%)	570 (47.2)	
Male sex, n (%)	667 (55.3)	
Marital status—married, n (%)	863 (71.5)	
Retired, n (%)	793 (65.7)	
**Comorbidities**		
Hypertension, n (%)	874 (72.4)	
Diabetes, n (%)	352 (29.2)	
COPD, n (%)	170 (14.1)	
CAD, n (%)	149 (12.3)	
Atrial fibrillation, n (%)	295 (24.4)	
Anemia (haemoglobin < 13 g/dL in men, < 12 g/dL in women), n (%)	406 (33.6)	
Renal dysfunction (eGFR < 60 mL/min/1.73 m^2^), n (%)	206 (17.1)	
**Stroke-related characteristics**		
CMG 108, n (%)	136 (11.3)	
CMG 109, n (%)	121 (10.0)	
CMG 110, n (%)	950 (78.7)	
Time from stroke onset to rehabilitation admission ≤ 30 days, n (%)	933 (77.3)	
Ischemic stroke, n (%)	971 (80.4)	
Haemorrhagic stroke, n (%)	236 (19.6)	
Dysphagia, n (%)	226 (18.7)	
Neglect, n (%)	170 (14.1)	
Aphasia, n (%)	525 (43.4)	
**Site of impairment**		
Right body, n (%)	602 (49.9)	
Left body, n (%)	605 (50.1)	
Motor-FIM score at admission, mean (SD)	18.6 (5.6)	
Cognitive-FIM score at admission, mean (SD)	17.1 (9.2)	
Total FIM score, mean (SD)	35.7 (13.0)	
**Laboratory findings** *		
Blood urea nitrogen (mg/dl), mean (SD)	20.9 (10.1)	
Serum creatinine (mg/dl), mean (SD)	0.89 (0.35)	
Estimated glomerular filtration rate (mL/min/1.73 m^2^), mean (SD)	83 (24)	
Serum sodium (mmol/l), mean (SD)	140.1 (5.5)	
Serum sodium < 135 mmol/l, n (%)	51 (4.2)	
Haemoglobin (g/dl), mean (SD)	13.2 (1.8)	
Total cholesterol (mg/dl), mean (SD)		

* Measured at admission to rehabilitation.

### Logistic regression

At multivariate analysis, age, diabetes, CAD, AF, anemia, renal dysfunction, neglect, and cognitive FIM score were significantly associated with 3-year mortality (Table 2). Age was the most important variable (Table 3).

**Table 2:** Results of the multivariate logistic regression analysis: beta (β) coefficients with standard deviations (SD), odds ratios with the 95% confidence intervals (CI) and the p-values are presented.

Variable	β coefficients (SE)	Odds Ratio (95% CIs)	P-value
Age (per 5-year increase)	0.269 (0.048)	1.31 (1.19–1.44)	0.000
Diabetes	0.352 (0.179)	1.42 (1.00–2.02)	0.050
History of CAD	0.762 (0.224)	2.14 (1.38–3.32)	0.001
Atrial fibrillation	0.408 (0.184)	1.50 (1.05–2.16)	0.027
Anemia	0.339 (0.175)	1.40 (1.00–1.98)	0.053
Renal dysfunction (eGFR < 60 mL/min/1.73 m^2^)	0.439 (0.203)	1.55 (1.04–2.31)	0.031
Neglect	0.609 (0.234)	1.84 (1.16–2.91)	0.009
Cognitive FIM score (per 1-point increase)	− 0.053 (0.011)	0.95 (0.93–0.97)	0.000

**Table 3:** Top-ranked variables in the logistic regression.

Variable	χ^2^	Likelihood ratio test p value
Age	56.83	0.0000
Cognitive FIM score	80.86	0.0000
History of CAD	95.54	0.0001
Neglect	103.07	0.0061
Renal dysfunction (eGFR < 60 mL/min/1.73 m^2^)	108.90	0.0158
Time from stroke occurrence to rehabilitation admission	113.47	0.0325
Diabetes	117.46	0.0457

The logistic model had an AUC of 0.745 (95% CI: 0.709–0.782). The Hosmer–Lemeshow χ^2^ was 9.48 (p value 0.303). Cox proportional hazard regression analysis was also computed as a further comparison and provided comparable results; the Cox model had a C index of 0.747 (95% CI 0.712–0.782) and was well calibrated (Hosmer–Lemeshow χ^2^ 8.57).

At the optimal risk threshold of 21% (Youden index 0.368), the logistic model had a sensitivity of 57.7% (95% CIs 50.3–64.8), a specificity of 79.1% (95% CIs 76.4–81.5), an accuracy of 75.7% (95% CIs 73.2–78.1), a PPV of 33.9% (95% CIs 28.7–39.3), and a NPV of 91.0% (95% CIs 88.9–92.7). Supplementary table S1 displays sensitivity, specificity, PPV, NPV, and accuracy of the model at various risk thresholds ranging from 5 to 50%.

### Machine learning algorithms

Table 4 shows the performance metrics of the ML algorithms before and after SMOTE application on the test data. The algorithms with SMOTE application clearly outperformed the algorithms without SMOTE application.

**Table 4:** Measures of performance with 95% confidence intervals for the machine learning-based algorithms before and after the implementation of SMOTE on the test data.

Algorithm	SMOTE	Sensitivity	Specificity	Accuracy	F-measure	AUC
RF	Not applied	0.422 (0.395–0.451)	0.904 (0.886–0.913)	0.763 (0.738–0.786)	0.510	0.844 (0.806–0.882)
GB	Not applied	0.465 (0.437–0.493)	0.888 (0.869–0.905)	0.764 (0.739–0.787)	0.535	0.810 (0.768–0.852)
ADA-B of RF	Not applied	0.516 (0.488–0.544)	0.879 (0.859–0.896)	0.773 (0.748–0.796)	0.571	0.870 (0.835–0.905)
RF	Applied	0.879 (0.854—0.900)	0.842 (0.815–0.865)	0.861 (0.844–0.876)	0.863	0.928 (0.902–0.954)
GB	Applied	0.841 (0.814–0.864)	0.863 (0.837–0.885)	0.852 (0.834–0.867)	0.850	0.927 (0.900–0.953)
ADA-B of RF	Applied	0.891 (0.866–0.911)	0.822 (0.794–0.846)	0.857 (0.839–0.872)	0.861	0.910 (0.880–0.939)

While the differences were small, the RF algorithm achieved the highest AUC and the highest F measure, which is a measure of a test's accuracy calculated based on the precision and recall, among the three algorithms with SMOTE application. The SMOTE RF model achieved an AUC of 0.928 (95% CIs 0.902–0.954) and an F-measure of 0.863. Sensitivity was 87.9% (95% CIs 85.4–90.0), and specificity 84.2% (95% CIs 81.5–86.5). Accuracy, that is, the proportion of both true positives and true negatives correctly identified, was 86.1% (95% CIs 84.4–87.6). As regards the parameters of the SMOTE RF model, the optimization loop of Knime analytics platform allowed us to obtain the best ones: the information gain ratio was used as split criterion, 100 trees were used, the maximum node size was one.

**Figure 2:**
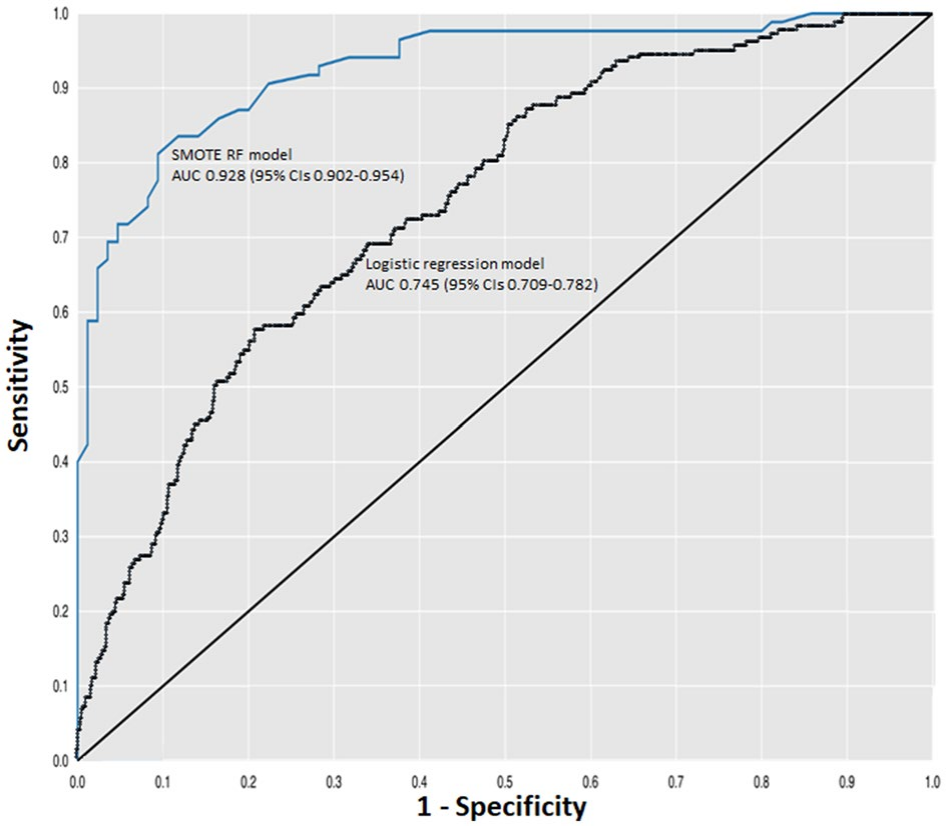
Receiver operating characteristics curves for the SMOTE RF algorithm and the logistic model.

The goodness of fit test was applied to calibrate the model and understand whether observed sample frequencies differ significantly from expected frequencies; the p-value of the chi square was equal to 0.605, proving the goodness of the SMOTE RF model. The PPV was 84.6% (95% Cis 82.4–86.5) and the NPV 87.5% (95% CIs 85.5–89.2). The Receiver Operating Characteristics curve for the SMOTE RF model and the multivariable logistic regression model are shown in Fig. 2. The SMOTE RF model clearly outperformed the logistic regression model. Of note, the ADA-B of RF (the parameters were the same of SMOTE RF model) was the best ML model without SMOTE and even this model was able to outperform the logistic regression one with an AUC of 0.870, a sensitivity of 51.6%, a specificity of 87.9% and an accuracy of 77.3%.

**Figure 3:**
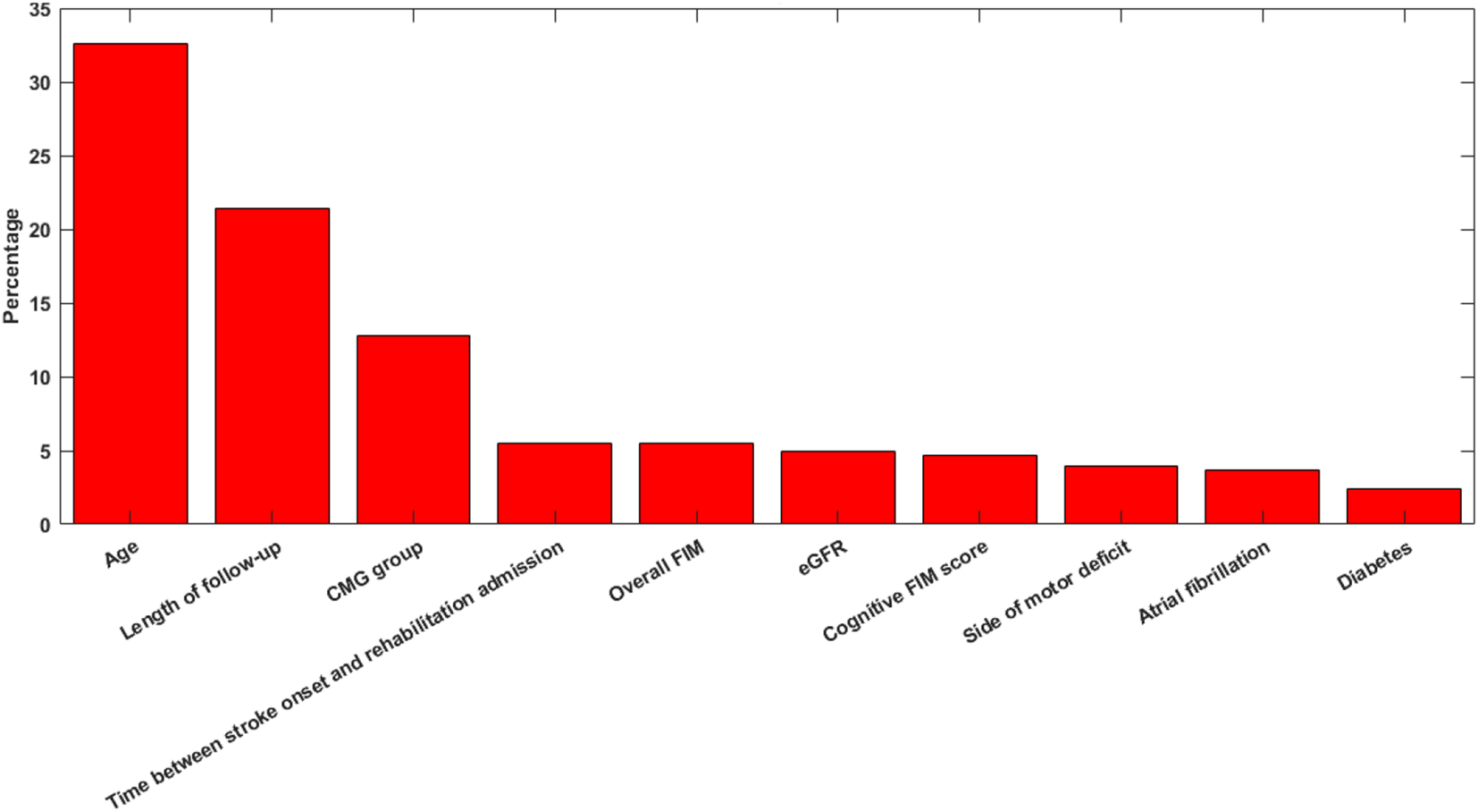
Top 10 features according to the SMOTE RF model.

The features importance according to the SMOTE RF algorithm was computed and is represented in Fig. 3. Age was the most important features.

In order to further confirm the findings from the features importance, the 10 most important features represented in Fig. 3 underwent also a univariate statistical analysis. A Kolmogorov Smirnov test, which is appropriate for large datasets, was performed to investigate the normality of the data (all p-values < 0.0001). Then, a Mann Whitney or a chi square tests were performed, and the results are shown in Table 5.

**Table 5:** Univariate statistical analysis of the most importance features identified by the SMOTE RF model.

Variables	Survivors	Deceased	p-value
Age, mean (SD)	69.15 (11.88)	77.92 (8.84)	< 0.001’
Length of the follow-up (days), mean (SD)	1762 (1192)	1258 (1117)	< 0.001’
**CMG, %**			
108	39.7	60.3	
109	79.3	20.7	< 0.001***^***
110	74.1	25.9	
Time from stroke onset to rehabilitation admission (days), mean (SD)	21.9 (15.52)	27.9 (18.39)	< 0.001’
Total FIM score, mean (SD)	36.9 (13.24)	32.8 (12.14)	< 0.001’
eGFR (mL/min/1.73 m^2^), mean (SD)	85 (24)	79 (26)	0.002’
Cognitive FIM score, mean (SD)	17.9 (9.5)	15.2 (8.5)	< 0.001’
Right side of motor deficit, (%)	50.5	48.4	0.522^
Atrial fibrillation, (%)	21.0	32.9	< 0.001^
Diabetes, (%)	26.2	36.3	< 0.001^

’ = Mann Whitney. ^ = Chi square.

Excluding the side of motor deficit (whose percentage is balanced between the two groups), all the other variables, indicated as relevant by the features importance analysis, had also highly statistical significant difference between the two groups, thus confirming again the valuable quality of the model.

## Discussion

Machine learning methods have gained increasing popularity in the setting of biomedical research. Machine learning-based algorithms may be used for screening, diagnostic, or prognostic purposes. In cardiovascular medicine, ML methods have been tested in several medical conditions to predict a future health state. The aim of this study was two-fold: to assess the relative performance of ML-based algorithms, with or without SMOTE application, for predicting long-term mortality in stroke patients with severe disability and to compare the performance of ML algorithms to that of a standard logistic regression model. There are three major findings of this study:

1. ML algorithms outperformed the standard logistic model for predicting 3-year mortality;
2. After SMOTE implementation, ML algorithms exhibited excellent overall performance, outperforming the algorithms without SMOTE application;
3. While the differences were small, the RF algorithm exhibited the best performance among the SMOTE algorithms.

The standard logistic model had moderate discriminatory value (AUC 0.745) and was well calibrated. This finding is in line with previous studies performed to develop prognostic models for 1-year mortality in patients with acute stroke (C statistics ranging from 0.71 to 0.84)^27,28^. Conventionally, AUC values > 0.70 are considered to represent moderate discrimination, values > 0.80 good discrimination, and values > 0.90 excellent discrimination. Nam et al. investigated the predictors of long-term mortality in 3,278 patients with acute ischemic stroke^29^. The cumulative death rate within 3 years was 18.4% and the model had a C index of 0.78^29^. While discrimination and calibration are essential properties of any prognostic model, they are uninformative as to clinical value. What a clinician needs to know is the proportion of the patients who will die or survive correctly identified^30^. According to Pfeiffer and Gall^31^, the concept of “concentration of risk” (i.e., the proportion of individuals who will develop the event of interest and who are included in the proportion of individuals with a risk exceeding a certain threshold) is more directly relevant to decision making. At the optimal risk threshold of 21% for 3-year mortality, the logistic model identified approximately six in ten patients who subsequently died as being at high risk, implying that 40% of the patients who died were not correctly classified as being at high risk. At the optimal risk threshold, the PPV was as low as 31%, implying that the proportion of false positives largely exceeded that of true positives.

All ML algorithms achieved better metrics of performance than the standard logistic model, with AUC in the range of 0.810 to 0.928. In cardiovascular prognostic studies, datasets often have an unequal class distribution, resulting in unbalanced dataset. This problem is known as imbalanced classification^32^. The SMOTE, though not exempt from intrinsic limitations, is a well-known data pre-processing technique to cope with imbalanced classification^32^. In this study, application of SMOTE did allow improve the predictive performance of ML algorithms. Notably, discrimination exceeded 0.90 after SMOTE application. Among the SMOTE algorithms, the RF algorithm appeared to have the best performance, as judged by discrimination and F-measure that is a measure of a test’s accuracy^33^. The SMOTE RF model achieved an AUC of 0.928 and an F-measure of 0.863. The high predictive performance of the SMOTE RF model was further confirmed by high sensitivity, specificity, and positive and negative predictive values while the goodness of the model was confirmed also by the univariate statistical analysis (9 features over the top 10 were statistically significant) that enforced the selection of features performed by the algorithms. The SMOTE RF algorithm had a sensitivity of 0.879 and a specificity of 0.842, meaning that the algorithm correctly identified 88% of the patients who died and 84% of the survivors. The PPV, that is, the probability that a patient will die when classified as being at high risk, was 0.846, implying that the proportion of false positives was as low as 15%. On the other hand, the NPV was 0.875, implying a very low proportion of false negatives. These findings suggest that ML methods can offer improvement over traditional regression models in predicting outcome.

Unsurprisingly, given that aging is characterized by increased vulnerability to death, age emerged as the most important predictor in both the standard logistic model and the SMOTE RF model. The deleterious changes at molecular, cellular, physiological, and functional levels that characterize aging in conjunction with the rapid shrinking or failure of compensatory and antagonistic responses to such changes may be the biological basis of increased vulnerability to death of aged patients^34^.

In conclusion, our findings suggest that the use of ML methods may offer improvement over traditional regression models in identifying stroke patients who are at risk of death. Assessing whether the improvement in prognostication achieved with ML methods translates into improved decision-making and clinical care remains an ongoing challenge.

## Limitation

There are some limitations in this analysis. First, despite having good results also on the unbalanced dataset, the use of SMOTE is a potential limitation for the study; having a balanced dataset would be helpful for this type of studies. Nevertheless, SMOTE is efficient to deal with unbalanced classes without giving up on having a large dataset^35^. Second, our ML analysis was fully addressed to a tree-based approach. While other classifiers can be employed, a fully tree-based approach and, in general, decision tree-based algorithms have already shown in literature their great potential^36–38^. Third, despite having performed a validation internally through the cross-validation, the models were not externally validated in an independent dataset and thus overfitting cannot be ruled out. Finally, although ML algorithms can be advantageous over traditional regression methods to predict prognosis, their implementation in clinical practice can be complicated. Apart from methodological issues, developing “patient-centered and clinician-friendly” ML-based predictive tools, assessing their potential contribution to clinical care and their reproducibility in health care remain major ongoing challenges^39–41^.

## Material and methods

### Participants

Patients were recruited from the specialized stroke rehabilitation units of the Maugeri IRF of Cassano Murge (Bari—Puglia), Telese Terme (Benevento—Campania), and Montescano (Pavia—Lombardia) in Italy. All data were extracted from the electronic Hospital Information System networked between the participating centers. Vital status was ascertained by linking with the regional Health Information System.

Enrolment periods varied among the participating centers but ran from February 2002 to September 2016 overall. A total of 3646 patients admitted for stroke rehabilitation were identified using a computer-generated list obtained from our administrative database and by reviewing electronic medical records. We included patients admitted to the participating IRFs ≤ 90 days from stroke occurrence and classified as CMG 0108 (weighted Functional Independence Measure [wFIM] motor score < 26.15 and age > 84.5), 0109 (wFIM motor score > 22.35 and < 26.15, and age < 84.5), or 0110 (wFIM motor score < 22.35 and age < 84.5) of the Medicare case-mix classification system^25^, who completed rehabilitation. Patients classified as CMGs 0101 to 0107 or admitted to rehabilitation > 90 days from stroke occurrence (N 2164), discharged against medical advice (N 92), for whom time from stroke occurrence to rehabilitation admission was not recorded (N. 40), or who did not complete rehabilitation (N 109), were excluded. One thousand two hundred forty-one patients fulfilled the selection criteria.

The Medicare classification system distinguishes 10 CMGs for stroke rehabilitation. Patients are assigned into one of the ten distinct CMGs, based on age, the sum of weighted ratings for 12 FIM-motor items (transfer to tub or shower item is excluded), and the sum of FIM cognitive ratings^25^. The FIM is currently the most widely used measure to describe the degree of impairment in activities of daily living in clinical practice. The motor-FIM score consists of 13 items assessing four domains of function (self-care, sphincter control, transfers, and locomotion). The cognitive-FIM score consists of five items assessing two domains (communication and social cognition). Each item is scored on a 7-point Likert scale, from 1 (total dependence) to 7 (total independence). The study was approved by the Institutional Review Board of the “Istituti Clinici Scientifici Maugeri” of Bari. Patients’ data were deidentified. Since the research was retrospective and did not present any risk of harm to subjects and the dataset did not contain identifying information, written informed consent was deemed to be unnecessary by the Institutional Review Board of the “Istituti Clinici Scientifici Maugeri” of Bari. All the procedures were performed according to the declaration of Helsinki.

### Definitions

Comorbidities were defined as described in a previous study^42^. Coronary artery disease (CAD) was diagnosed based on a documented history of myocardial infarction, percutaneous coronary angioplasty, or coronary artery bypass grafting, or a previous hospitalization for CAD. Renal dysfunction was defined as estimated glomerular filtration rate < 60 mL/min/1.73 m^2^. Anemia was defined as haemoglobin less than 12 g/dL in women and less than 13 g/dL in men. Atrial fibrillation (AF) was diagnosed based on admission electrocardiogram. Chronic obstructive pulmonary disease (COPD) was diagnosed based on patient's medical records documenting a past diagnosis of COPD, chronic medication used for COPD, and/or previous hospitalizations for exacerbation of COPD. The Bedside Swallowing Assessment Scale, administered by a trained speech therapist, was used to diagnose dysphagia. If concerns regarding the safety and efficiency of swallow function emerged from the scale, a fiberoptic endoscopic evaluation of swallowing was performed. The Semi-Structured Scale for the Functional Evaluation of Hemi-inattention was used to diagnose personal neglect.

### Logistic regression model and statistical analysis

Data are reported in the following sections as mean and standard deviation for continuous variables or percentage for categorical variables. The covariates examined included age (per 5-year increase), marital status (married/not married), hypertension, diabetes, COPD, history of CAD, AF, anemia, renal dysfunction, time from stroke onset to rehabilitation admission, ischemic stroke, dysphagia, neglect, and motor and cognitive FIM scores at admission. These variables were selected based on prior studies showing an association with the outcomes of interest^6,30,42–56^. A multivariate logistic regression analysis with backward stepwise selection (p > 0.20 for exclusion) was performed to assess the association of covariates with 3-year mortality. We examined the strength and shape of the relations of continuous variables with the log odds of death including nonlinear terms and using cubic spline technique. Odds ratios with their 95% confidence intervals (CIs) and β coefficients were calculated. The model was internally validated by resampling 200 bootstrap replications. Discrimination was assessed using the area under the receiver operating characteristics area under the curve (AUC). Calibration was assessed using the Hosmer–Lemeshow test. The importance of each variable was measured by using a likelihood ratio test. Finally, we calculated sensitivity, specificity, accuracy, positive predictive value (PPV), and negative predictive value (NPV) of the optimal risk threshold identified by using maximum value of the Youden index^57^. The primary outcome was all-cause mortality up to 3 years from discharge from rehabilitation.

### Machine learning: tools and algorithms

The Knime Analytics Platform (version 3.7.1) was used for variable selection and the implementation of the algorithms. The Knime Analytics Platform version 3.7.1 was chosen since it is a well-known analytics platform already used in previous studies^58,59^ and it resulted as the best choice for advanced users in a comparison with other platforms and programming languages^60^. It allows the users to create workflows of ML analyses by combining nodes and is integrated with other software, thus allowing other researchers a high reproducibility of the analysis. Three tree-based ML model algorithms were performed: random forests (RF), ADA-Boost (ADA-B), and gradient boosting (GB). The Synthetic Minority Over-sampling Technique (SMOTE) was used to cope with imbalanced classification.

### Synthetic minority over-sampling technique

The Synthetic minority oversampling technique (SMOTE) is an important algorithm that is applied to balance the different number of examples of each class^35^. It produces artificial data by picking between a real object of a specified class and one of its nearest neighbours (of the same class). Subsequently, it selects a point along the line between these two objects determining a new one**.**

### K-fold cross-validation

A tenfolds cross-validation was applied to compute the evaluation metrics on all the ML models. It is a resampling procedure used to evaluate machine learning models. The procedure has a single parameter called k that consists in the number of groups that a given dataset is split into. The metrics were computed on the best subset of features obtained through the wrapper, employing the tenfolds cross-validation^61^. This workflow allows obtain the best subset of features for the analysed patients and limit overfitting since the wrapper is computed with a tenfold cross-validation^62^.

### Tree-based algorithms and their evaluation

Tree-based algorithms are empowerments of a simpler decision tree that can make it stronger and let it achieve higher accuracy in the prediction tasks^36–38^. They belong to the so-called supervised learning, which consists in making a classifier learn from the data by providing it with the classes of each subject. In this research, the input data were both categorical and nominal features while the output/target of the analysis was the categorical variable “deceased/survivor”.

We used the wrapper for variable selection. It selects the best subset of variables in a given dataset, which maximizes the accuracy of the predictions.

Several classifiers can be used and are well-known in literature; among all we chose to follow a tree-based approach because in literature it has often been successful in literature^63–65^ and, particularly, because the decision tree (J48), a well-known structure made up of leaves and nodes that represent the features and the classes, allowed us to open the black box nature of ML algorithms by performing the top 10 feature importance and, consequently, the univariate statistical analysis on those features. Each split in the tree can be performed in different ways, the most used (that also give similar results when applied) being information gain and gini index^66^. The empowered versions considered in this study were: Gradient Boosted tree (GB), Random Forests (RF) and Ada-boosting (ADA-B) of RF^67–69^. Each of them uses one of the ensemble learning techniques to improve the model of J48: randomization, bagging and boosting. RF is an example of bagging and randomization: aiming to make the model variance decrease, bagging trains each tree of the forest using a randomly drawn subset of features using the patients of the training set. The employment of bagging is particularly useful to limit overfitting; thus, RF results extremely powerful in limiting overfitting^57^. To make a prediction on a new patient, RF aggregates predictions from all their decision trees by a majority vote. ADA-B uses only the clinical features that allow obtain a higher accuracy and a lower mathematical complexity for the model. Moreover, it builds an ensemble by adding a new model that emphasizes the training instances that previous models misclassified. In this paper, the hyperparameter configuration was performed through an optimization loop node that is available in Knime analytics platform.

The following evaluation metrics were used to evaluate model performance:

- Sensitivity = TP/(TP + FN),
- Specificity = TN/(TN + FP),
- Accuracy = (TP + TN)/(TP + FN + TN + FP),
- PPV = TP/(TP + FP),
- NPV = TN/(FN + TN),
- Area under the Receiver Operating Characteristics curve (AUC),

where TP denotes true positives, FP false positives, TN true negatives, and FN false negatives.

To evaluate the performance and efficiency of the ML based model, the F-measure was also calculated^70^. For F-measure, the maximum is 1. F-measure is calculated as the harmonic mean between recall and precision values, where the former indicates the portion of positive patterns that are correctly detected while the latter indicates the positive patterns that are correctly identified from the overall predicted patterns in a positive group. A high accuracy with low F-measure and specificity or sensitivity indicates an unbalanced dataset that could require the implementation of SMOTE to balance positives and negatives.

Finally, the calibration of the model was tested through a goodness of fit test which is employed to verify whether sample data fits a distribution from a certain population, in this case to understand how well the actual(observed) data points fit into our ML models.

## Data availability 

The datasets generated during and/or analysed during the current study are not publicly available due to privacy policy but are available from the corresponding author on reasonable request.

## Supplementary information

Supplementary Table S1.
