# Supplementary material for: Machine learning to predict mortality after rehabilitation among patients with severe stroke
Source: Sci Rep. 2020 Nov 18;10:20127. doi: 10.1038/s41598-020-77243-3 (PMC7674405; doi:10.1038/s41598-020-77243-3)
Supplement: Supplementary file 1 — Supplementary Table S1. [file 41598_2020_77243_MOESM1_ESM.pdf]

Machine learning to predict mortality after rehabilitation among patients with severe stroke

Authors and affiliations:

Domenico Scrutinio[a], Carlo Ricciardi[b,a], Leandro Donisi[b,a], Ernesto Losavio[a], Petronilla Battista[a], Pietro Guida[a], Mario Cesarelli[ca], Gaetano Pagano[a], Giovanni D'Addio[a].

a) Istituti Clinici Scientifici Maugeri IRCCS, Pavia, Italy.
b) Department of Advanced Biomedical Sciences, University Hospital of Naples "Federico II", Naples, Italy
c) Department of Electrical engineering and information technology, University of Naples "Federico II", Naples, Italy.

***Corresponding author:**

Carlo Ricciardi

Department of Advanced Biomedical Sciences

University Hospital of Naples "Federico II", Italy

Mobile: +39 3282572629

Supplementary table S1. Sensitivity, specificity, accuracy, positive predictive value, and negative predictive value of the logistic regression model at various risk thresholds.

Risk thresholds	TP (N)	FN (N)	TN (N)	FP (N)	Sensitivity	Specificity	Accuracy	PPP	PPN
>5%	181	8	229	789	95.8 (91.8-98.2)	22.5 (20.0-25.2)	34.0 (31.3-36.7)	18.7 (16.3-21.3)	96.6 (93.5-98.5)
>10%	166	23	472	546	87.8 (82.3-92.1)	46.4 (43.3-49.5)	52.9 (50.0-55.7)	23.3 (20.3-26.6)	95.4 (93.1-97.0)
>15%	134	55	638	380	70.9 (63.9-77.3)	62.7 (59.6-65.7)	64.0 (61.2-66.7)	26.1 (22.3-30.1)	92.1 (89.8-94.0)
>20%	110	79	775	243	58.2 (50.8-65.3)	76.1 (73.4-78.7)	73.3 (70.7-75.8)	31.2 (26.4-36.3)	90.7 (88.6-92.6)
>25%	87	102	859	159	46.0 (38.8-53.4)	84.4 (82.0-86.6)	78.4 (75.9-80.7)	35.4 (29.4-41.7)	89.4 (87.3-91.3)
>30%	63	126	913	105	33.3 (26.7-40.5)	89.7 (87.7-91.5)	80.9 (78.5-83.0)	37.5 (30.2-45.3)	87.9 (85.7-89.8)
>35%	45	144	961	57	23.8 (17.9-30.5)	94.4 (92.8-95.7)	83.3 (81.1-85.4)	44.1 (34.3-54.3)	87.0 (84.8-88.9)
>40%	28	161	985	33	14.8 (10.1-20.7)	96.8 (95.5-97.8)	83.9 (81.7-86.0)	45.9 (33.1-59.2)	86.0 (83.8-87.9)
>45%	18	171	1001	17	9.5 (5.7-14.6)	98.3 (97.3-99.0)	84.4 (82.3-86.4)	51.4 (34.0-68.6)	85.4 (83.3-87.4)
>50%	13	176	1010	8	6.9 (3.7-11.5)	99.2 (98.5-99.7)	84.8 (82.6-86.7)	61.9 (38.4-81.9)	85.2 (83.0-87.1)

Abbreviations. N denotes the number of patients, TP true positives, FP false positives, TN true negatives, FN false negatives, PPV positive predictive value, and NPV negative predictive value.
